# Micro-CT vs. Whole Body Multirow Detector CT for Analysing Bone Regeneration in an Animal Model

**DOI:** 10.1371/journal.pone.0166540

**Published:** 2016-11-23

**Authors:** Oliver Bissinger, Jan S. Kirschke, Florian Andreas Probst, Martin Stauber, Klaus-Dietrich Wolff, Bernhard Haller, Carolin Götz, Christian Plank, Andreas Kolk

**Affiliations:** 1 Department of Oral- and Maxillofacial Surgery, Klinikum rechts der Isar der Technischen Universität München, Munich, Germany; 2 Department of Diagnostic and Interventional Radiology, Section of Neuroradiology, Klinikum rechts der Isar der Technischen Universität München, Munich, Germany; 3 Department of Oral- and Maxillofacial Surgery, Ludwig-Maximilians-University of Munich, Munich, Germany; 4 Scanco Medical AG, Brüttisellen, Switzerland; 5 Institute of Medical Statistics and Epidemiology, Klinikum rechts der Isar der Technischen Universität München, Munich, Germany; 6 Institute of Molecular Immunology – Experimental Oncology, Klinikum rechts der Isar der Technischen Universität München, Munich, Germany; University of Sheffield, UNITED KINGDOM

## Abstract

**Objectives:**

Compared with multirow detector CT (MDCT), specimen (ex vivo) micro-CT (μCT) has a significantly higher (~ 30 x) spatial resolution and is considered the gold standard for assessing bone above the cellular level. However, it is expensive and time-consuming, and when applied in vivo, the radiation dose accumulates considerably. The aim of this study was to examine whether the lower resolution of the widely used MDCT is sufficient to qualitatively and quantitatively evaluate bone regeneration in rats.

**Methods:**

Forty critical-size defects (5mm) were placed in the mandibular angle of rats and covered with coated bioactive titanium implants to promote bone healing. Five time points were selected (7, 14, 28, 56 and 112 days). μCT and MDCT were used to evaluate the defect region to determine the bone volume (BV), tissue mineral density (TMD) and bone mineral content (BMC).

**Results:**

MDCT constantly achieved higher BV values than μCT (10.73±7.84 mm^3^ vs. 6.62±4.96 mm^3^, p<0.0001) and consistently lower TMD values (547.68±163.83 mm^3^ vs. 876.18±121.21 mm^3^, p<0.0001). No relevant difference was obtained for BMC (6.48±5.71 mm^3^ vs. 6.15±5.21 mm^3^, p = 0.40). BV and BMC showed very strong correlations between both methods, whereas TMD was only moderately correlated (r = 0.87, r = 0.90, r = 0.68, p < 0.0001).

**Conclusions:**

Due to partial volume effects, MDCT overestimated BV and underestimated TMD but accurately determined BMC, even in small volumes, compared with μCT. Therefore, if bone quantity is a sufficient end point, a considerable number of animals and costs can be saved, and compared with in vivo μCT, the required dose of radiation can be reduced.

## Introduction

In our study, we used an established CSD (critical-size defect) model in the jaw of a rat, as previously described [1]. Several studies have evaluated the bone regeneration of CSDs. Most studies employ conventional radiology for visualising bone regeneration, μCT for quantification, histology and histomorphometry to describe the (sub-)cellular microarchitecture, and immunohistochemistry with antibodies to stain structures such as blood vessels [2, 3].

Ex vivo μCT determines several three-dimensional (3D) indices concerning the bone microarchitecture, leading to a better estimation of its quality and quantity. However, it is a relatively expensive technique and allows only the assessment of endpoints [4].

In contrast, in vivo μCT (= preclinical μCT) allows longitudinal in vivo measurements. However, fast scan speeds are only possible at intermediate and low resolution. At its best resolution, the scanning time and radiation dose increase considerably, and the volume of interest (VOI) is restricted [5]. Nevertheless, for in vivo applications, the scan speed is highly important [6]. Since the development of MDCT, the spatial resolution and imaging speed have improved substantially, and an in-plane spatial resolution of 250 μm is now achievable in the clinic [6–9]. Although the spatial resolution is lower in MDCT compared with preclinical μCT (approximately 250 μm × 250 μm × 500 μm vs. 10 μm isotropic), MDCT has the advantages of being widely available and capable of visualising trabecular structures in humans [10].

In a recently published study, Bauer et al. used trabecular bone specimens and have shown that both μCT and MDCT are able to accurately predict the failure load (FEM and biomechanical testing were performed) at spatial resolution, which is available in vivo (up to 250 μm × 250 μm × 500 μm). The authors considered this resolution to be the optimal compromise between accuracy and a fast scan time [10].

In another previously published study evaluating the bone structure of the calcaneus, significant correlations between the measures of the bone volume fraction (BV/TV) obtained using μCT and MDCT were found [11]. The objective of these studies was the non-invasive prediction of fracture risk in osteoporosis patients [12].

Despite the large number of indices, BV (or BV/TV) is the most widely accepted index for the assessment of bone regeneration [13]. Therefore, we wanted to investigate whether the BV or a surrogate measure can be determined using MDCT to quantify bone regeneration.

As the scanning time (several minutes) and the radiation exposure of in vivo μCT scans is known to be higher (272–1088 mGy depending on the resolution; [5, 14] and assessments by the authors) compared with MDCT (scanning time: seconds, radiation exposure: 25 mGy) and as several effects of radiation exposure in terms of sequential in vivo μCT analyses like decreased trabecular bone volume are already described [5, 15, 16], the aim of our study was to examine whether the lower resolution of the MDCT is sufficient to qualitatively and quantitatively evaluate bone growth in the rat mandible.

As MDCT is widely available, these in vivo follow-up examinations would allow many researchers to save a large number of animals and financial resources in bone regeneration studies.

## Materials and Methods

### Animal model

The animal experiment was authorised by the local animal research committee (Regierung von Oberbayern) in accordance with German legislative requirements and was performed at the Institute of Experimental Oncology and Therapy Research, Centre for Preclinical Research at the Technical University of Munich (reference number: 55.2-1-54-2531-100/03).

Forty six-month-old male Sprague Dawley (SD) rats (weight range: 479–501 g) obtained from Charles River WIGA (Sulzfeld, Germany) were taken from another study (the largest group) evaluating the in vivo dose dependency of a non-viral vector and were acclimatised for at least 2 weeks. Because every animal used for this study received the same therapy (BMP-2 plasmid), we did not expect an influence on the measurements performed in this study. Vendor health reports indicated that the rats were free of known viral, bacterial and parasitic pathogens. According to the standards for animal housing, the rats were singly fed, housed at 23–25°C (55±5% humidity with a 12-h light/dark cycle), and allowed free access to water and standard laboratory pellets. The animals’ health status was monitored throughout the experiments by a health surveillance programme according to Federation of European Laboratory Animal Science Associations (FELASA) guidelines.

The rats were preoperatively anaesthetised with Medetomidine (Medetomin, 1.0 mg/ml, Dechra Veterinary Products, ‘s-Hertogenbosch, Netherlands), Midazolam (Midazolam, 5 mg/ml, Hexal AG, Germany) and Fentanyl (Fentadon, 50 μg/ml, Dechra Veterinary Products, ‘s-Hertogenbosch, Netherlands) via intramuscular (i.m.) injection.

**Fig 1:**
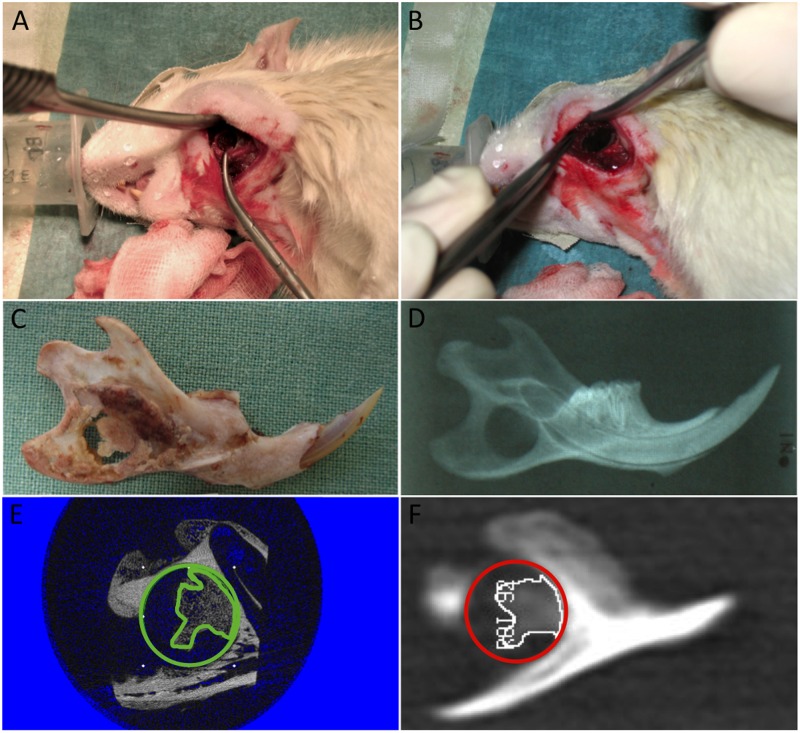
Surgical procedure. (A) A transosseous critical-size defect (CSD) of 5 mm in diameter was burred into the area of the jaw angle of the mandible, and then, (B) the mandibular CSD was covered with a coated bioactive 8-mm Ø titanium disk. Clinical (C) and radiographic (dental X-ray, D) images of the same specimen reveal the newly formed woven bone after 14 days. The corresponding 2D μCT (E) and MDCT (F) images show the NB (colour edged) within the ROI (circle) of the CSD.

Non-healing full-thickness defects of 5 mm in diameter (CSDs) were created in the mandibular angle (Fig 1A, Table 1) [1]. The defects were covered with 8 mm-diameter coated bioactive titanium implants (BMP-2 plasmid) (Fig 1B). The copolymer-protected gene vectors (COPROGs) and poly (D,L-lactide) (PDLLA)-coated titanium discs used in this study were purchased and prepared as previously described [17].

**Table 1:** Study setup indicating the time points for death and the corresponding numbers of specimens.

Day	7	14	28	56	112
Number of rats	8x	8x	8x	8x	8x

Eight rats were evaluated per time point

At the end of the operation, an antidote combination composed of Atipamezole (Antisedan, 5 mg/ml, Orion Corporation, Espoo, Finland), Flumazenil (Flumazenil, 0.1 mg/ml, Hexal AG) and Naloxone hydrochloride (Naloxone, 0.4 mg/ml, Braun AG, Germany) was administered subcutaneously (s.c.).

During the postoperative period, pain was relieved by subcutaneous administration of buprenorphine twice a day (Buprenodale, 0.3 mg/ml, Dechra Veterinary Products), and all efforts were made to minimise suffering. Before the rats were sacrificed on days 7, 14, 28, 56 and 112 (group size per time point: 8) with an overdose of Narcoren (sodium pentobarbital, 80 mg/kg BW), they were anaesthetised in a plastic box via isoflurane inhalation. The time points in this study were chosen based on the time course of rat fracture healing [18, 19]. All mandibles were freed of soft tissue and the titanium disc (Fig 1C), fixed in 100% methanol and stored at 4°C (overnight). Plain X-ray images of the mandibles were acquired (Fig 1D) using a dental X-ray machine (Gendex Corporation, Des Plaines, IL, USA) as a standard procedure to determine whether the defect had a proper and comparable localisation. Prior to 3D scanning, the specimens were positioned parallel to the scanning beam (rigidly attached to a match) for both modalities to get the same sagittal slice to determine the region of interest (ROI).

### Micro CT

The mandibles were scanned using a μCT system (μCT 40, Scanco Medical, Bruttisellen, Switzerland) with a nominal resolution of 8 μm. The μCT was operated at an energy of 55 kVp and an intensity of 145 μA, which corresponds to a focal spot size of 5 μm, capable of producing cone beams detected by a 12 bit charge-coupled device (CCD) detector (2048 x 64 pixels). A total of 1000 projections was taken over 180°, with an integration time of 200 ms per projection (Average Data: 1). The samples were fixed perpendicular (to the length of the sample holder) in an airtight sample holder filled with methanol, to prevent them from drying during the measurement (field of view = FOV: 16.4 mm). These tubes were marked with an alignment line on the outside of the sample holder to allow the consistent positioning of the specimens. An alignment notch served as a reference point to connect the sample holder with the turntable; this arrangement permitted the mandible to be positioned exactly within seconds.

As this specific μCT system can scan a maximum of 208 slices per stack, 2 stacks had to be measured for each sample. On average, 302 slices, with a total height of 2.416 mm, were acquired. The total examination time per specimen was approximately 30–50 minutes [20].

A standard convolution-backprojection procedure with a Shepp and Logan filter was used to reconstruct the 3D CT images into matrices of 2048 x 2048 voxels per slice.

Attenuation values were converted to hydroxyapatite (HA) concentrations using a linear model (density calibration) obtained from scans of a hydroxyapatite (HA) phantom provided by the manufacturer of the system. The phantom consisted of five 6-mm-diameter HA cylinders of known density (0, 100, 200, 400 and 800 mg HA/cm^3^), for which 0 mg HA/cm^3^ represented a soft tissue equivalent background devoid of mineral. To correct for beam hardening artefacts, a correction algorithm based on a 1200 mg HA/cm^3^ wedge phantom was applied.

**Fig 2:**
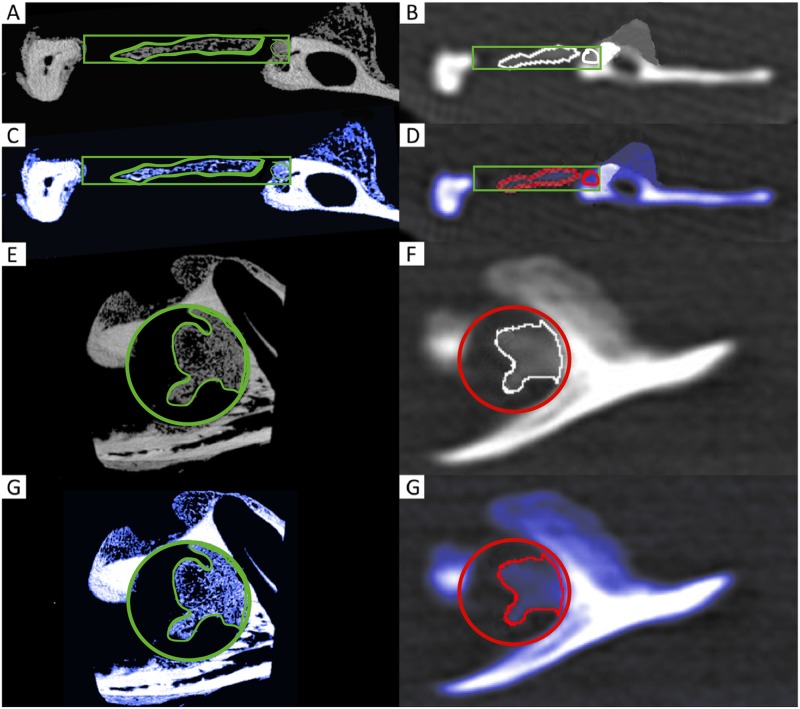
2D coronal (A—D) and sagittal (E—H) μCT (left column) and MDCT (right column) images (A, B, E and F) and corresponding colour-coded illustrations (C, D, G and H) of the CSD region. The newly formed woven bone is shown in dark (blue), and the original cortical bone is shown in bright (white). Within the former cylindrical defect of 5 mm (VOI), the ROI is illustrated.

A standard circle with a diameter of 5 mm corresponding to the defect was manually drawn and served as the ROI (Fig 1E). A VOI (height, approximately 2.42 mm; diameter, 5 mm), which corresponded to the maximum medial-lateral width of the defect depending on the amount of bone regeneration, was determined from the 2D images (Figs 1E and 2 left column). A constrained 3D Gaussian filter was used to partially suppress the noise in the volumes. A global threshold was determined visually by two independent examiners (based on slice-wise 2D comparisons between the grey scale and segmented image of all samples and the associated histograms) [5, 7, 21]. All samples were binarised using the same parameters for sigma (1.2), support (2), and threshold (353.5 mg HA/cm^3^) [22]. All image processing steps were conducted automatically using Image Processing Language (IPL, Scanco Medical, Bruttisellen, Switzerland) on an Alpha-based open VMS workstation (DS20E, Hewlett Packard, Inc.) [22, 23].

The following measures were evaluated from the μCT image data for each specimen: bone volume (BV, mm^3^), tissue mineral density (TMD, mg HA/cm^3^), and bone mineral content (BMC, defined as the callus BV multiplied by TMD, mg) [23, 24]. Two-voxel “peeling” was used when calculating the TMD to minimise partial volume effects [24, 25]. The bone regeneration indices obtained from these data sets were used as the gold standard for comparison with the indices derived from MDCT [7].

### Multirow detector CT

Images for the bone analysis were acquired using a 16-row MDCT scanner (Sensation 16; Siemens Medical Solutions, Erlangen, Germany). Samples were scanned rigidly fixed in the same position in the sample holder as for μCT (parallel to the scanning beam). A high-resolution protocol was used, which was similar to the protocol applied in the clinic for high-resolution inner ear measurements. The tube current was 120 kVp with 300 mAs/slice and a scan time of approximately 12 seconds/sample. This protocol had an in-plane spatial resolution of 210 x 210 x 500 μm (pixel size: 100 x 100 x 500 μm). It had a collimation and a table feed of 0.5 mm and a reconstruction index of 0.5 mm. The images were reconstructed with the two high-resolution kernels “U80 u very sharp” and “U90 u ultra sharp” at a very small field of view of 50 mm and an image matrix size of 512 x 512 pixels. For calibration purposes, a reference phantom (Osteo Phantom, Siemens) was placed below the specimens. The images were transferred to a Leonardo workstation (Siemens Medical Solutions, Erlangen, Germany) and reformations were acquired with the multiplanar reformation tool (MPR; visually matched to the orientation of the μCT) using the same slice thickness as the original images. In the first step of the image analysis, the MDCT images were binarised: therefore, we applied an optimised global threshold to all images (70 mg HA/cm^3^). This threshold was visually optimised to distinguish dense and sparse bone formation, as described previously [21]. Due to the substantial partial volume effects in case of MDCT, this threshold had to be chosen lower as compared to μCT. This hydroxyapatite threshold (THRCa) was converted to Hounsfield units (HUs) for every image, as previously described [21]. After binarising, the images with this threshold, ROIs were selected within the sagittal section in the same inclination according to the μCT (Figs 1F and 2 right column). Textural analysis was performed with software developed in-house using IDL (Creaso, Gilching). The TMD and BMC values were calculated using the calibration phantom. The morphological indices obtained from the MDCT corresponded with the μCT indices [7, 21, 26].

### Statistical analysis

All data are presented as mean values ± standard deviations (means ± SD). Using Pearson’s correlation coefficients, we examined the association between the μCT and MDCT results. Additionally, a linear regression model was fitted to the data, with the MDCT data as the dependent variable and the μCT data as the independent variable. Regression coefficients and the root mean squared error (RMS) are presented. Differences between modalities were assessed by the paired t-test. Bland-Altman plots showing the difference of the measurements versus the mean of the measurements with a line for the mean difference and 95% limits of agreement (LoA) were used to assess the agreement of the modalities [27]. All statistical analyses were conducted using GraphPad Prism Version 6.00 (GraphPad Prism Software^®^ San Diego, CA, USA). For all tests, P < 0.05 was considered significant.

## Results

MDCT was used to quantitatively evaluate measures of bone to detect the temporal differences in the BV, TMD and BMC of the new bone (NB) and to assess the correlations with the values derived from μCT, which was used as the gold standard.

The border of the (former) defect to the existing bone was evident, and the structure and brightness were clearly distinct between the existing bone and new bone, enabling an unproblematic positioning of the circle in both modalities to determine the ROI (Figs 1 and 2).

**Fig 3:**
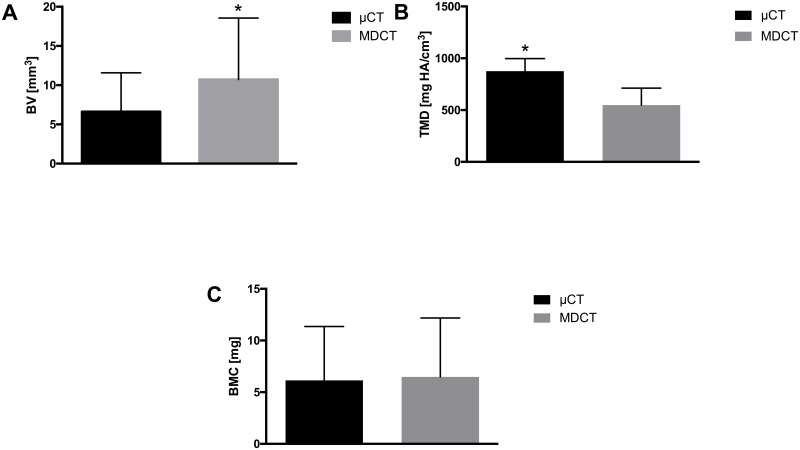
A shows the significantly higher BV values obtained with MDCT compared with μCT, whereas B shows the significantly lower TMD values obtained. The BMC values matched well between μCT and MDCT (C, p = 0.05). The data represent means ± SDs; the notation * indicates significance at p < 0.0001 between the modalities.

**Fig 4:**
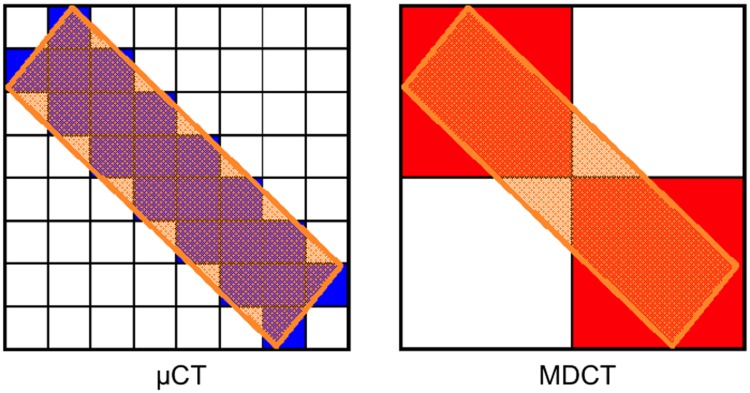
Yellow rectangle representing the bone to be measured (left: μct, right: MDCT). Voxels that are filled to more than 50% are counted as bone (blue or red), while those that are filled to less than 50% are not counted (white). Because of the lower resolution of the larger voxels of the MDCT, it generates a higher BV than that of the real bone. In contrast, the μCt, with its high resolution and very small voxel size, generates a more precise volume.

MDCT significantly overestimated the BV values compared to μCT (10.73 mm^3^ ± 7.84 vs. 6.62 mm^3^ ± 4.96, mean of the differences = bias: + 4.11 mm^3^, p < 0.0001, Fig 3A, Table 2). In contrast, the MDCT data significantly underestimated the TMD values compared to μCT (547.68 mg HA/cm^3^ ± 163.83 vs. 876.18 mg HA/cm^3^ ± 121.21, bias: - 328.5 mg HA/cm^3^, p < 0.0001, Fig 3B, Table 2). Differences in the quality of resolution (Fig 2) between μCT (left column) and MDCT (right column) are clearly visible in a scheme explaining the influence of the different voxel sizes (Fig 4): voxels that were filled to more than 50% with mineralised tissue were counted as bone (blue = μCT, red = MDCT), whereas those that were filled to less than 50% with mineralised tissue were not counted (white). The yellow rectangle representing the bone to be measured (left: μCT, right: MDCT) was imaged much more precisely with μCT than with MDCT.

**Table 2:** Mean values and differences between the MDCT-based and μCT-based parameters within the former CSDs, as well as the corresponding p-values of the t-test and Pearson’s correlation coefficients.

Parameter	MDCT (Mean)	μCT (Mean)	Mean difference [Limits of agreement]	t-test p-value	Pearson’s correlation
BV	10.73	6.62	+ 4.11 [-12.54–4.32]	< 0.0001*	0.87 p < 0.0001*
TMD	547.68	876.18	– 328.50 [92.67–564.30]	< 0.0001*	0.68 p < 0.0001*
BMC	6.48	6.15	+ 0.33 [-5.14–4.48]	0.40	0.90 p < 0.0001*

The MDCT-based BV (mm^3^) was significantly higher than the corresponding μCT data. The μCT-based TMD (mg HA/cm^3^) was consistently higher than the corresponding MDCT data. Similar BMC values (mg) were observed for both the MDCT-based and corresponding μCT-based data. Entries marked with * represent significant differences (p < 0.001).

In contrast to BV and TMD, the absolute BMC values agreed well between MDCT and μCT (6.48 mg ± 5.71 vs. 6.15 mg ± 5.21, bias: + 0.33 mg, p = 0.40, Fig 3C, Table 2).

**Fig 5:**
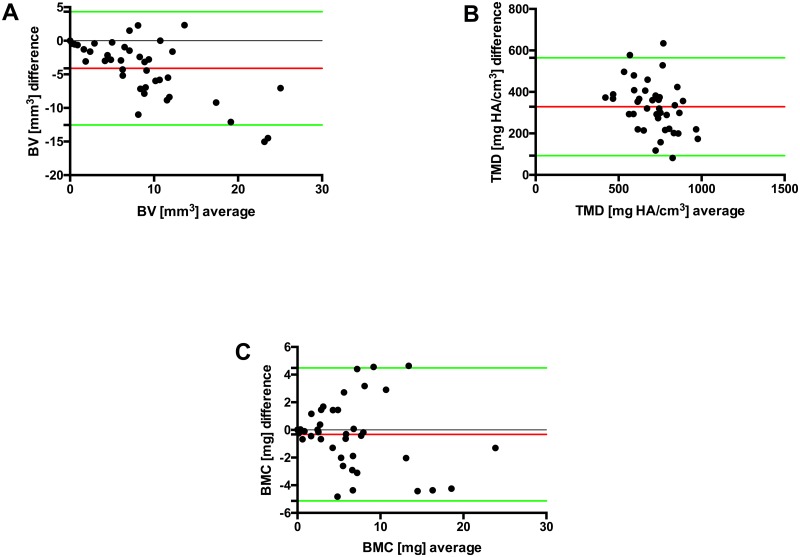
Bland-Altman plot of the difference (y-axis) between the measurements obtained by the two methods against their mean value (x-axis). The red line displays the mean difference, representing the bias between the methods. The green lines indicate the limits of agreement (mean of the differences ± 1.96 x SD of the differences).

The bias is shown in the Bland-Altman plot (Table 2, Fig 5) as the mean difference (red line, deviation from 0) between the measurements obtained by MDCT and μCT, which represents the systematic error. The limits of agreement (LOA, mean of the differences ± 1.96 x SD of the differences) are shown as green lines. The spread of the deviations from the mean of the differences is displayed by the values within the limits of agreement (BV: ± 8.43 mm^3^, TMD: ± 328.49 mg HA/cm^3^; BMC: ± 4.81 mg) (Table 2).

**Fig 6:**
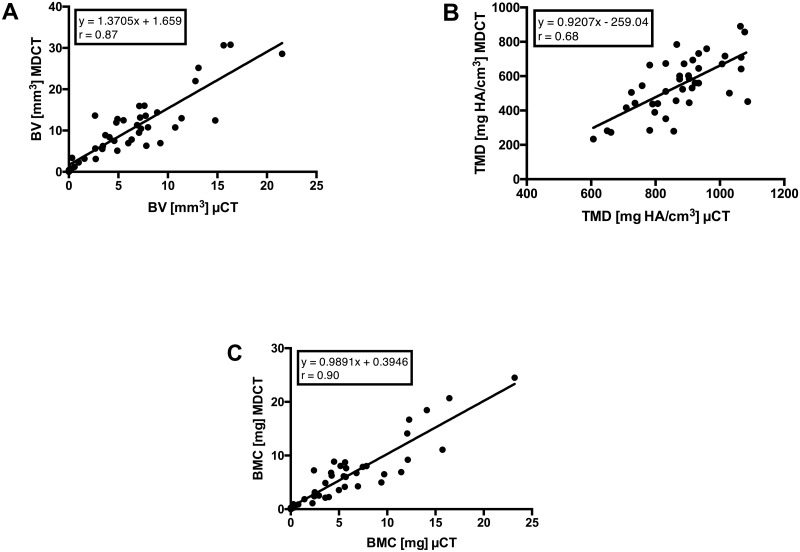
Scatterplots with a regression line, correlation coefficient (r), and estimated regression model showing the associations for BV (A), TMD (B) and BMC (C) obtained via μCT and MDCT (p < 0.0001).

BV showed a very strong correlation between the two methods (r = 0.87, p < 0.0001, Fig 6A), whereas TMD was moderately correlated between the methods (r = 0.68, p < 0.0001, Fig 6B). BMC yielded the strongest correlation between MDCT and μCT (r = 0.90, p < 0.0001, Fig 6C). The estimated regression equations for BV, TMD and BMC were y = 1.37x + 1.66 (RMS = 3.94 mm^3^), y = 0.92x – 259.04 (RMS = 121.5 mg HA/cm^3^), and y = 0.99x + 0.39 (RMS = 2.49 mg), respectively.

In summary, MDCT consistently generated higher BV values over time, with a very strong correlation with μCT, whereas it consistently showed lower TMD values, with a moderate correlation with μCT. The BMC values obtained using μCT and MDCT did not show a relevant difference but exhibited the strongest correlation.

## Discussion

In vivo and ex vivo μCT has a significantly higher spatial resolution than MDCT and is the gold standard in assessing bone density and structure above the cellular level. MDCT is characterised by its lack of invasiveness and very short scan times [4, 7, 22, 28]. Therefore, the objective of this study was to investigate whether the lower resolution of MDCT is sufficient to qualitatively and quantitatively determine the bone regeneration of a CSD in the rat mandible following stimulation with bioactive titanium implants. In summary, we found that MDCT was sufficient to accurately evaluate the development of bone growth with regard to BMC.

In detail, MDCT consistently generated higher BV values over time compared with μCT. This result was caused by partial volume effects attributed to the lower resolution of MDCT with larger voxel sizes (Figs 4 right, 3A, 5A and 6A). In contrast, the absolute μCT BV values reveal the correct volume because of its high resolution and very small voxel size (Fig 4 left). However, there was a very strong correlation between the methods; therefore, the bias (of significant overestimation) is correctable using the respective regression equation because of the functional relationship between MDCT and μCT. Regarding the TMD, the μCT results showed consistently higher levels than the corresponding MDCT data and only a moderate (but significant) correlation (Figs 3B, 5B and 6B). The bias of the higher μCT values was also correctable using the respective regression equation. In contrast to BV and TMD, no relevant differences in BMC were observed between the modalities, with a strong correlation between them. Here, the partial volume effects are much less pronounced, as the errors in TMD and BV are inversely related to each other and thus reduced in the BMC measurements. (Figs 3C, 5C and 6C).

The SD of the differences of BV was 4.30 (TMD: 120.3); therefore, the differences were smaller than ± 8.43 (TMD: 328.49) in 95% of the samples. This difference represents a relevant spread of the deviations from the mean of the differences and cannot be regarded as tolerable when comparing the absolute values of different studies. The spread of the BMC deviations was considerably lower (2.45), and the differences were in 95% of the samples smaller than 0.33 ± 4.81. Therefore, the effect of the methods on BMC was low and quite uniform, such that the absolute BMC values of exclusive MDCT measurements will not differ relevant from the μCT-based BMC values, and, thus, might not change the conclusions between bone regeneration studies. However, in studies evaluating effects of pharmaceutical therapies, differences can be considerably smaller so that these might not be detected with MDCT. In general, comparisons within studies and between studies seem to be feasible.

Despite the large number of 3D indices, such as BV, TV, BMD/TMD, BMC, trabecular indices, cortical indices and marrow volume, and in accordance with the results of our study, most authors of bone regeneration studies confine themselves to quantitative indices, such as BV, BMC or BV/TV, to establish their results [1, 13, 29–34].

The introduction of high-resolution peripheral quantitative computed tomography (HR-pQCT) enabled trabecular structures to be visualised in vivo [35]. Although the spatial resolution is lower in whole body MDCT compared with HR-pQCT or flat-panel CT (XperCT) (approximately 250 μm × 250 μm × 500 μm vs. 40 μm isotropic vs. 100 μm isotropic), MDCT has the advantages of being widely available and capable of visualising trabecular structures in the spine and proximal femur of humans [36]. However, no structural indices have been assessed (MDCT) in our rat model because of the smaller trabeculae in rats [37].

Similar to the previous tomographs, cone-beam in vivo μCT enables sequential in vivo measurements, and in contrast to MDCT, it offers a resolution very close to that of μCT. However, the ionising radiation (especially in high resolution mode) to which the animals are exposed in follow-up studies steadily accumulates and might lead to undesirable effects on the tissues that can potentially influence the results [5, 30]. The radiation dose in an in vivo μCT system (with similar settings to ours) has a range of 272 mGy to 1088 mGy, depending on the resolution and should be kept for in vivo longitudinal measurements below 500 mGy [5, 14]. This dose is assumed to be higher than the dose for MDCT (25 mGy) [15]. However, there is no study evaluating the MDCT dose on small samples such as ours. Standard dose calculations from MDCT cannot be used, as the probe scanned in this paper is much smaller than standard phantoms used for this purpose. The radiation exposure of ex vivo μCT (Scanco μCT 40) scans is known to be even higher (1980 mGy) [38]. Because of the theoretical character of our ex vivo study, our protocols were not optimised for a low radiation dose in MDCT or μCT.

Young, growing animals and proliferative biological processes might be particularly susceptible to radiation exposure. In addition, the radiation effects might be different depending on the tissue (tumour or regenerating bone vs. tendons), therapy (e.g., bioactive scaffolds seeded with stem cells vs. antibiotics) and age (e.g., growing bone vs. adult bone). Obviously, further studies are necessary to determine the possible effects of radiation exposure in sequential in vivo μCT analysis in a broad spectrum of research and to evaluate the radiation dose of MDCT on small specimens [5, 16, 30, 39]. Another potential limitation is the animal’s breathing cycle, particularly when scanning the axial skeleton and adjacent areas at high resolution for long scan times [30].

In our study, we have used an established CSD model in the rat jaw, as previously described [1, 13, 18], to ensure that our results are better comparable to those of other (following) studies. The model is considered economically advantageous, as the various stages of fracture healing are approximately twice as fast as those in humans [40]. Additionally, the mechanisms of fracture healing are similar, which makes this rodent an advantageous object for such research [41].

A close functional relationship exists between angiogenesis and osteogenesis. The presence of an adequate blood supply is a prerequisite for fracture healing or the integration of scaffolds and their replacement with bone [42]. Thus, in addition to bone, vessels are of interest in bone regeneration studies. For a CSD in the jaw, one study of rabbits using a lead-chromate-containing silicone rubber contrasting agent (Microfil MV-122, Flow Tech, Carver, MA, USA) has been reported [42]. However, as a limitation of the in vivo modalities, this technology is only available post mortem. Therefore, in addition to in vivo sequential measurements of the NB via in vivo μCT or MDCT, studies should be performed to establish ex vivo vessel μCT imaging (at the endpoint of a study) of the mandible of the rat after demineralisation to evaluate the vascular system after application of a bioactive scaffold. [30].

As a further limitation, our work has been performed on specimens (ex vivo). However, for in vivo applications, the surrounding soft tissues and skull bones must also be considered.

BV, TMD and BMC were significantly correlated between the two methods. However, because of partial volume effects, MDCT overestimated the bone volume and underestimated the bone density, although it accurately reflected the bone mass, even in small volumes. Therefore, in the future, sequential in vivo examinations seem to be feasible and rational because of the non-invasiveness, short scan times, accuracy for obtaining BMC, and low radiation of MDCT, and could be performed, based on our model. Consequently, if bone quantity is a sufficient end point, a considerable number of animals might be saved and costs could be reduced in future bone regeneration studies, as MDCT with this spatial resolution is widely available.

## Supporting Information

S1 File ARRIVE checklist. (PDF)

S2 File Editorial Certificate. (PDF)

S3 File Raw Data. (XLSX)
