# Supplementary material for: Micro-CT vs. Whole Body Multirow Detector CT for Analysing Bone Regeneration in an Animal Model
Source: PLoS One. 2016 Nov 23;11(11):e0166540. doi: 10.1371/journal.pone.0166540 (PMC5120815; doi:10.1371/journal.pone.0166540)
Supplement: S1 File — (PDF) [file pone.0166540.s001.pdf]

The ARRIVE Guidelines Checklist *

Animal Research: Reporting In Vivo Experiments

Carol Kilkenny[1], William J Browne[2], Innes C Cuthil[3], Michael Emerson[4], and Douglas G Altman[5]

[1] The National Centre for the Replacement, Refinement and Reduction of Animals in Research, London, UK, [2]School of Veterinary Science, University of Bristol, Bristol, UK, [3]School of Biological Sciences University of Bristol, Bristol, UK, [4]National Heart and Lung Institute, Imperial College London, UK, [5]Centre for Statistics in Medicine, University of Oxford, Oxford, UK

	Item	Recommendation	Section/ Paragraph
Abstract	1	Provide an accurate summary of the background, research objectives, including details of the species or strain of animal used, key methods, principal findings and conclusion of the study.	'Abstract-section'
Introduction			
Background	2	Explain how and why the animal species and model being used can address the scientific objectives and, where appropriate, the study's relevance to human biology.	'Introduction- and discussion-section'
Methods			
Ethical statement	3	Indicate the nature of the ethical review permissions, relevant licences (e.g. Animal [Scientific Procedures] Act 1986) and national or institutional guidelines for the care and use of animals, that cover the research.	'Material and Methods – animal model-section'
Study design	4	For each experiment, give brief details of the study design including:<br>a. The number of experimental and control groups.<br>b. Any steps taken to minimise the effects of subjective bias when allocating animals to treatment (e.g. randomisation procedure) and when assessing results (e.g. if done, describe who was blinded and when).<br>c. The experimental unit (e.g. a single animal, group or cage of animals).<br>A time-line diagram or flow chart can be useful to illustrate how complex study designs were carried out.	'Material and Methods – animal model-section'
Experimental procedures	5	For each experiment and each experimental group, including controls, provide precise details of all procedures carried out. For example:<br>a. How (e.g. drug formulation and dose, site frequency and route of administration, anaesthesia and analgesia used [including monitoring], surgical procedure, method of euthanasia). Provide details of any specialist equipment used, including supplier(s).<br>b. When (e.g. times of day).<br>c. Where (e.g. home cage, laboratory, water maze).<br>d. Why (e.g. rationale for choice of specific anaesthetic, route of administration, drug dose used).	'Material and Methods – animal model-section'
Experimental animals	6	a. Provide details of the animals used, including species, strain, sex, developmental stage (e.g. mean or median age plus age range) and weight (e.g. mean or median weight plus weight range).<br>b. Provide further relevant information such as the source of animals, international strain nomenclature, genetic modification status (e.g. knock-out or transgenic), genotype, health/immune status, drug or test naïve, previous procedures, etc.	'Material and Methods – animal model-section'

*Adapted from the ARRIVE guidelines. Originally published in PLoS Biology, June 2010[1]

Housing and husbandry	7	Provide details of:<br>a. Housing (type of facility e.g. specific pathogen free [SPF]; type of cage or housing; bedding material if relevant; number of cage companions; tank shape and material, etc. for fish).<br>b. Husbandry conditions (e.g. breeding program, light/dark cycle, temperature, quality of water, etc for fish, environmental enrichments).<br>c. Diet, including access to food and water, type of food (e.g. commercial source, product number), details of specialized diets if relevant (e.g. Ca-, P- levels, high-fat, -sucrose, -fructose).<br>d. Welfare-related assessments and interventions that were carried out prior to, during, or after the experiment.	'Material and Methods – animal model-section'
Sample size	8	a. Specify the total number of animals used in each experiment, and the number of animals in each experimental group.<br>b. Explain how the number of animals was arrived at. Provide details of any sample size calculation used.<br>c. Indicate the number of independent replications of each experiment, if relevant.	'Material and Methods – animal model- and statistical analysis-sections'
Allocating animals to experimental groups	9	a. Give full details of how animals were allocated to experimental groups, including randomisation or matching if done.<br>b. Describe the order in which the animals in the different experimental groups were treated and assessed.	'Material and Methods – animal model-section'
Experimental outcomes	10	Clearly define the primary and secondary experimental outcomes assessed (e.g. cell death, molecular markers, behavioural changes).	'Material and Methods – Micro CT-, Multi slice CT- and statistical analy
Statistical	11	a. Provide details of the statistical methods used for each analysis.<br>b. Specify the unit of analysis for each dataset (e.g. single animal, group of animals, single neuron).<br>c. Describe any methods used to assess whether the data met the assumptions of the statistical approach.	'Material and Methods – statistical analysis-section'
Results			
Baseline Data	12	For each experimental group, report relevant characteristics and health status of animals (e.g. weight, microbiological status, if relevant, and drug or test naïve) prior to treatment or testing. (This information can often be tabulated).	'Material and Methods – animal model-section'
Numbers analysed	13	a. Report the number of animals in each group included in each experimental group. Report absolute numbers (e.g. 10/20, not 50%[2]).<br>b. If any animals or data were not included in the analysis, explain why.	'Material and Methods – animal model- and Results-sections'
Outcomes and estimation	14	Report the results for each analysis carried out, with a measure of precision (e.g. standard error or confidence interval).	'Result-section'
Adverse events	15	a. Give details of all important adverse events in each experimental group<br>b.Describe any modifications to the experimental protocols made to reduce adverse events.	'Material and Methods – animal model- and results-section'
Discussion			
Interpretation/scientific implications	16	a. Comment on the study limitations including any potential sources of bias, any limitations of the animal model, and the imprecision associated with the results.	'Introduction- and Discussion-section'

NC 3Rs

References:

1. Kilkenny C, Browne WJ, Cuthill IC, Emerson M, Altman DG (2010) Improving Bioscience Research Reporting: The ARRIVE Guidelines for Reporting Animal Research PLoS Biol 8(6): e1000412. Doi:10.1371/journal.pbio.1000412
2. Schulz KF, Altman DG, Moher D, the CONSORT Group (2010) CONSORT 2010 Statement: updates guidelines for reporting parallel group randomised trials. BMJ 340:c332.
